# Nurse-led management of sialorrhea in Parkinson's disease: a pilot randomized controlled trial

**DOI:** 10.3389/fmed.2026.1788173

**Published:** 2026-03-19

**Authors:** Huimin Guan, Xiaolin Ma, Chuyue Jiang, Hui Liu, Yuling Xu, Guangjian Zhang, Heng Liu

**Affiliations:** 1 School of Nursing, Qingdao University, Qingdao, Shandong, China; 2 Department of Traumatology, Qingdao Municipal Hospital, Qingdao, Shandong, China; 3 Department of Pain, Affiliated Hospital of Yanbian University, Yanji, Jilin, China

**Keywords:** delphi method, nursing, Parkinson's disease, sialorrhea, symptom management theory

## Abstract

**Background:**

One of the most prevalent non-motor symptoms in people with Parkinson's disease (PD) is sialorrhea. In addition to aggravating pre-existing swallowing and speech issues, sialorrhea frequently causes perioral dermatitis, choking episodes, and aspiration pneumonia. In extreme situations, patients are forced to carry handkerchiefs at all times, which not only damages their reputation and results in social embarrassment and isolation, but also initiates or intensifies psychiatric symptoms like sadness and anxiety. Current management of PD-related sialorrhea includes pharmacological treatments, botulinum toxin, and behavioral strategies. While interventions such as speech therapy and oral exercises are effective, they are often applied episodically in clinical settings. Consequently, patients struggle to sustain these techniques daily. Addressing the lack of integrated nursing protocols for continuous home-based application, this study aimed to develop a structured sialorrhea management program based on the Theory of Symptom Management (SMT) and evaluate its feasibility and preliminary effectiveness.

**Methods:**

Based on SMT, an intervention was constructed via expert Delphi consultations. Subsequently, 80 patients with PD-related sialorrhea were randomized into control or intervention groups in a single-center trial. Efficacy was evaluated after four weeks based on sialorrhea severity and self-management capacity.

**Results:**

We created an intervention framework with 13 secondary indicators, 39 tertiary indicators, and three primary indicators (symptom experience, symptom management strategies, and symptom management results) after two rounds of Delphi expert consultation. Following the four-week intervention, the intervention group demonstrated significantly lower scores on the Sialorrhea Clinical Scale for PD and the sialorrhea-related item of the Unified PD Rating Scale than the control group (*P* < 0.05). Furthermore, the intervention group achieved a significantly higher score in self-management ability compared to the control group (*P* < 0.05).

**Conclusions:**

As a structured approach designed to eventually improve quality of life, this sialorrhea intervention program provides preliminary evidence of feasibility and early efficacy in reducing symptom severity and bolstering self-management.

**Clinical Trial Registration:**

https://www.chictr.org.cn/index.html, ChiCTR2500096034.

## Background

Parkinson's disease (PD) affects 1.37% of Chinese people 60 years of age and older, making it the second most common neurological disease after Alzheimer's disease (1). China is predicted by the World Health Organization (WHO) to have around 5 million PD patients by 2030, which would represent half of the world's PD population (2). The motor symptoms of PD include bradykinesia, muscle rigidity, resting tremor, and postural balance abnormalities (3), while the non-motor symptoms include sleep disturbances, autonomic dysfunction, cognitive impairment, and neuropsychiatric symptoms like depression and anxiety (4). While autonomic dysfunction in PD can also manifest as xerostomia (dry mouth), sialorrhea remains a more socially debilitating symptom that requires targeted intervention (5). About 32% to 74% of PD patients experience sialorrhea (6), which is primarily attributed to impaired oral-pharyngeal clearance and a reduced frequency of spontaneous swallowing, rather than true salivary hypersecretion (7). In addition to exacerbating pre-existing swallowing (dysphagia) and speech (dysarthria) issues, sialorrhea frequently causes perioral dermatitis, coughing/choking episodes, and aspiration pneumonia. The symptoms of sialorrhea get considerably worse as PD advances. Patients in severe cases need to use handkerchiefs constantly, which not only impairs their self-esteem and leads to social shame and isolation, but it also causes or exacerbates anxiety and sadness, which together place a heavy physical and mental load on patients (8). Beyond the clinical impact on patients, the chronic nature of PD imposes a substantial burden on caregivers, who frequently experience role strain, fatigue, and social stigma (9). This burden extends significantly to the family's economic stability and the broader healthcare system (10). In China, the financial toll is particularly severe; research indicates that the average annual disease-related cost constitutes nearly 44.8% of a family's household income (11). Such high expenditures not only deplete family resources but also pose critical challenges to the sustainability of elderly care services in an increasingly aging society (12). Given these multifaceted challenges, there is an urgent need for cost-effective intervention strategies targeting both motor and non-motor symptoms to alleviate the cumulative strain on patients, families, and the healthcare infrastructure.

Current treatment interventions for PD-related sialorrhea have serious limits, despite the condition's widespread and complex effects. Pharmacological therapies such anticholinergic drugs, injections of botulinum toxin, or surgery are the mainstay of traditional methods (13). The majority of studies, however, focuses on specific therapy methods, frequently ignoring the wider, complex impact of drooling on a patient's life, even if comprehensive and varied rehabilitation regimens are necessary (14). They frequently overlook the substantial psychological distress, social shame, and reduced quality of life that people endure, which results in interventions that may lack a truly patient-centered focus and sufficiently integrated perspective. Furthermore, the overall effectiveness and patient satisfaction of these traditional approaches may be limited due to their potential for side effects, lack of sustainability, and infrequent consideration of the patient's particular circumstances, preferences, or the shifting nature of Parkinson's symptoms (15).

**Figure 1:**
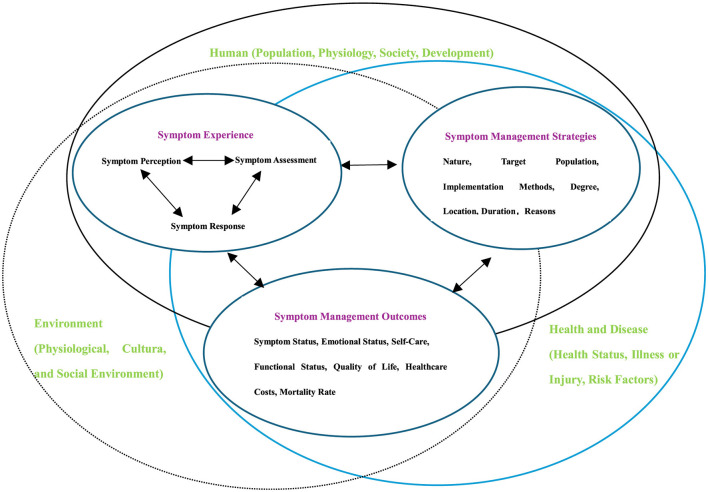
Theoretical framework diagram. Framework diagram of symptom management theory.

To address this gap, this study developed and evaluated an intervention framework grounded in the Symptom Management Theory (SMT), specifically tailored for sialorrhea in PD patients. Developed by the University of California, San Francisco School of Nursing, SMT incorporates the fundamental nursing concepts of person, environment, health, and illness (16, 17) into a robust model for patient-centered care. The theory posits that effective management requires addressing three interrelated dimensions: symptom experience (the patient's perception and distress), management strategies (behavioral and nursing interventions), and symptom outcomes (measurable results). By adopting this framework, the current intervention connects these dimensions specifically for PD sialorrhea (Figure 1) (18). This theoretical underpinning ensures that the program transcends a mere collection of exercises, offering instead a holistic approach aligned with the patient's subjective experience (19).

SMT has emerged as a prominent framework in nursing research. Originally developed to address symptom burden in cancer care, SMT has demonstrated robust efficacy across diverse chronic conditions, including traumatic brain injury and chronic obstructive pulmonary disease (COPD). Since its systematic introduction to China in 2012 (20), the theory has been instrumental in promoting evidence-based nursing, proving that structured, theory-driven interventions significantly enhance patients' self-efficacy and quality of life (21).

However, despite its proven versatility, the application of SMT to PD-related sialorrhea remains largely unexplored. This represents a critical gap, as sialorrhea is a multifaceted issue involving not only physical dysfunction but also significant psychological distress (e.g., embarrassment) and behavioral challenges. SMT is uniquely suited to address this complexity because it moves beyond passive medical treatment. By integrating patients' subjective symptom experiences with actionable management strategies, SMT empowers individuals to actively modify relevant behaviors—such as swallowing frequency and posture—within their daily environment, thereby providing a theoretically grounded and practical foundation for developing targeted self-management interventions.

Guided by this theoretical fit, the primary objective of this study was to construct and evaluate an SMT-based intervention framework specifically tailored for PD sialorrhea. The study focuses on alleviating symptom distress, while the intervention incorporates structured behavioral training designed to facilitate treatment adherence, thereby potentially contributing to improved quality of life by fostering patient self-management capabilities.

## Methods

Guided by SMT, this study adopted the methodological framework established by Geng et al. (22) for participant recruitment, data collection, and statistical analysis, while ensuring independent execution and unique data generation. Initially, a systematic literature review and Delphi expert consultations were conducted to finalize the intervention protocol. Subsequently, a two-arm, parallel-group randomized controlled trial was carried out at the neurology department of a tertiary hospital between July and September 2025. This study employed an assessor-blinded design. While participant blinding was precluded by the behavioral nature of the intervention, potential detection bias was minimized through a strict separation of roles: outcome assessors were not involved in care delivery and remained blinded to allocation. Similarly, the statistician analyzed masked datasets to ensure objectivity. Furthermore, to minimize attrition bias, the statistical analysis followed the Intention-to-Treat (ITT) principle, ensuring that all 80 randomized participants were included in the final evaluation regardless of their level of adherence.

### Establishing a study group

The research team comprised nine multidisciplinary members: one postgraduate supervisor, two graduate nursing students, two registered nurses, one clinical physician, one rehabilitation therapist, one psychologist, and one nutrition expert. Seven of these members (the supervisor, two students, two registered nurses, the physician, and the rehabilitation therapist) are listed as authors based on their substantial contributions to the study's execution and manuscript preparation. The remaining two specialists (psychologist and nutrition expert) served as technical consultants and are recognized in the acknowledgments. This study was approved by the Ethics Committee of Qingdao University Medical College (QDU-HEC-2024407), and all participants provided written informed consent before enrollment.

To ensure methodological rigor and maintain the nurse-led focus, specific roles were assigned as follows:

Project Conception and Data Analysis: The postgraduate supervisor and the lead graduate nursing student were responsible for the study design, final statistical analysis, and data interpretation.

Delphi Process: The two graduate nursing students coordinated the systematic literature review and managed the expert recruitment and correspondence for the two-round Delphi process.

Patient Recruitment: The two registered nurses, in collaboration with the clinical physician, were responsible for participant screening and recruitment within the neurology department.

Intervention Delivery (Nurse-Led): Consistent with the nurse-led design, the daily delivery of the 30-min intervention sessions was performed exclusively by two designated registered nurses.

Outcome Assessment: To ensure a strictly blinded design, outcome assessments were conducted by two independent clinical nurses from the neurology department who were not members of the core nine-person research team and remained unaware of group assignments.

Technical Support: The physician, rehabilitation therapist, and consultants (psychologist and nutritionist) collaborated to design and validate the specialized training modules (e.g., medical safety, swallowing protocols, and psychosocial support) prior to their implementation by the registered nurses.

### Literature search and analysis

Researchers looked through Web of Science, PubMed, Embase, JBI, Up to Date and Parkinson Net for pertinent material between January 2019 and October 2024. By looking for a combination of subject phrases and free words, the fundamental structure of the intervention program was established.

The literature was chosen based on the following criteria: research on sialorrhea in PD that was published within the previous five years; examples of this type of literature include guidelines, expert consensus, systematic reviews, cross-sectional studies, retrospective studies, and randomized controlled trials. Non-English/Chinese literature, duplicate publications, research with inadequate literature evaluation, and the lack of a full text were the criteria used to remove the material. The Australian JBI Center for Evidence-Based Health Care's literature quality evaluation tool for expert consensus, systematic reviews, and randomized controlled trials, as well as the AGREE II evaluation tool for guidelines, were used to evaluate the quality of the included literature.

After consulting with the research team, two graduate students conducted a two-person quality evaluation, extracting and summarizing relevant data on reducing sialorrhea symptoms in PD patients both domestically and abroad. They then created the initial draft of the intervention protocol.

### Delphi's knowledgeable reaction

#### The development of the expert correspondence questionnaire

The expert correspondence questionnaire was created based on the literature review and the study's objectives after discussion within the research group. There are four main sections to it: (1) Form instructions, outlining the study's history, the form's guidelines, and the due date for questionnaire returns. (2) Basic information from experts. (3) The “SMT” and the “Sialorrhea” are introduced, together with definitions of pertinent theories and concepts. (4) Program consultation with three level one, fifteen level two, and fifty-six level three entries, covering symptom experience, symptom management tactics, and symptom management outcomes.

#### Selecting the professionals to consult

Experts in clinical nursing, neurology, rehabilitation, psychology, and nutrition were chosen through the technique of purposive sampling. Requirements for inclusion: (1) People who have worked in clinical practice, clinical nursing, nursing management, or PD-related nursing education for ten years or more. (2) Possess at least a bachelor's degree. (3) Hold a title of associate senior or above. (4) Take part in and direct this study voluntarily.

Based on the predetermined inclusion criteria, 25 potential experts were initially identified through professional networks and a systematic literature review. These candidates were contacted via email or telephone to explain the study's purpose and requirements. Five candidates declined participation due to time constraints or personal reasons, and no experts were excluded during the initial screening as all remaining candidates met the inclusion criteria. Consequently, 20 specialists provided informed consent. Ultimately, the 16 specialists who completed the consultation process were included in the final Delphi panel, representing nine provinces and cities: Hubei, Shaanxi, Liaoning, Beijing, Shanxi, Jiangsu, Anhui, Henan, and Jilin. Of these, 12 were nursing-related professionals, while the remaining panel members included one specialist each in neurodegenerative diseases, rehabilitation, psychology, and nutrition.

The final composition of the panel was intentionally weighted toward nursing (75%) because the intervention is fundamentally a nurse-led management program grounded in the nursing-specific SMT. This distribution ensured that the intervention strategies were feasible and applicable within clinical nursing workflows. However, to ensure multidisciplinary rigor and clinical safety, specialists in neurodegenerative diseases (for diagnostic accuracy), rehabilitation (for swallowing training protocols), psychology (for emotional support strategies), and nutrition (for dietary guidance) were included. This balance allowed the research team to integrate medical, rehabilitative, and nutritional guardrails into a nursing-centered framework.

#### Using expert correspondence

From June to August 2024, there were two rounds of expert consultation for this study, and the questionnaires were sent out by email or paper. After the first round of expert consultation, the research team prepared the second round of expert consultation questionnaire after reviewing and revising the entries and contents in response to the experts' feedback. Questionnaires were sent out every two to three weeks until the experts' views aligned. Questionnaire entries with importance and feasibility ratings of 3.5 or lower, as well as coefficients of variation more than 0.25, were eliminated. The research team then discussed and updated the corresponding entries, taking into consideration the thoughts and recommendations of the experts.

### Study participants

Participants were recruited from the Department of Neurology at a tertiary A hospital in Qingdao who have been diagnosed with PD-related sialorrhea. They underwent screening by attending neurologists to determine their eligibility to take part in the experiment. Inclusion criteria included: (1) PD diagnosis in compliance with the Diagnostic Criteria for PD in China (2016 Edition) is a prerequisite for inclusion. (2) Drooling is indicated by a score of ≥ 1 on item 6 (sialorrhea) of the Unified PD Rating Scale Part II (UPDRS-II). (3) Giving written informed consent and being willing to take part in the study willingly. Exclusion Criteria: (1) Patients with Parkinson-plus syndromes or subsequent Parkinsonism are excluded. (2) Individuals with psychiatric illnesses, severe vision or hearing impairments, or severe cognitive impairment. Dropout criteria include: (1) non-compliance or voluntarily leaving the study. (2) The emergence of a clinical condition that is unstable and hinders ongoing involvement. (3) Missing more than two weeks in a row of follow-up appointments.

This study was prospectively registered with the Chinese Clinical Trial Registry (ChiCTR) under the registration number ChiCTR2500096034 (https://www.chictr.org.cn/index.html). Written informed consent was obtained from the participants before their participation in the study.

### Sample size calculation

The sample size was determined based on data from our preliminary pilot study, with the Sialorrhea Clinical Scale for PD (SCS-PD) score serving as the primary outcome measure. Using a two-sided significance level (α) of 0.05 and a statistical power (1–β) of 0.80, a projected effect size (Cohen's *d*) of 0.78 was anticipated, based on an expected mean difference of 2.5 points in SCS-PD scores between groups. These parameters indicated that a minimum sample of 33 participants per group was required. To compensate for an anticipated attrition rate of approximately 20%, we enrolled 40 participants per group, resulting in a total final sample size of 80.

### Randomization and blinding

This study used an allocation concealment approach to avoid allocation bias. An impartial third party used a computer-generated random number table to assign groups. Prior to participant enrollment and assignment, the allocation sequence was kept secret by using opaque, sealed envelopes. The control and experimental groups were kept at a 1:1 allocation ratio. To ensure consistency in intervention delivery and minimize inter-therapist variability, the interventions for both groups were administered by the same team of two registered nurses. However, to prevent contamination, the nurses underwent rigorous training to strictly separate the protocols: they followed a standardized script for general health education in the control group, while strictly adhering to the SMT-based program for the experimental group. To mitigate participant expectancy bias (specifically the placebo effect), participants were informed that the study aimed to compare two distinct, active nursing management models, rather than a “treatment vs. no-treatment” comparison.

### Group allocation and intervention procedures

After providing voluntary written informed consent, subjects were randomly assigned to two groups. Both groups continued to receive standard routine care provided by the ward staff. To control for the potential effects of professional attention (attention bias), both groups received an additional 30-min session daily, five days per week, for four weeks, delivered by the designated research nurses.

Control Group: Patients in this group received a structured general health education program. The curriculum covered general PD management topics, including: (1) Medication adherence (e.g., timing of levodopa doses); (2) Home safety and environment (e.g., fall prevention); (3) General nutritional guidance (e.g., protein-levodopa interaction); and (4) Sleep hygiene and basic psychological reassurance (e.g., managing anxiety). Sessions were conducted via verbal instructions and standardized pamphlets. Crucially, to differentiate this from the experimental intervention, strictly no information regarding oral-motor exercises, swallowing compensatory techniques, or drooling-related psychosocial coping strategies was provided to this group. Experimental Group: In addition to standard routine care, this group received an individualized sialorrhea intervention based on the SMT framework. At baseline, each patient's symptom experience (severity, frequency, and distress) was assessed to tailor specific management tactics. The program comprised four core modules: (1) Targeted health education (e.g., saliva management and hygiene); (2) Psychosocial support (addressing embarrassment and anxiety); (3) Oral-motor exercises (e.g., lip and tongue strengthening); and (4) Swallowing training (e.g., frequency cueing and compensatory postures). While the total session time was fixed at 30 min to match the control group, the intensity and focus of the exercises were dynamically adjusted based on the patient's progress and individual condition.

### Intervention implementation and quality control

The intervention was primarily nurse-led and followed a multidisciplinary design. While the protocols and weekly supervision were provided by a multidisciplinary team (including a rehabilitation therapist and a psychologist), the daily 30-min sessions were delivered by two trained registered nurses to ensure consistency. The program comprised mandatory core components (oral-motor exercises and hygiene education) and individualized strategies (e.g., targeted psychosocial support or posture training) tailored to each patient's specific SMT assessment.

To ensure intervention fidelity, all sessions were documented using a standardized checklist within a nursing manual, and weekly team meetings were conducted to monitor and resolve any protocol deviations. Participant adherence was assessed via the session completion rate, defined as the percentage of the 20 planned sessions (5 days per week for 4 weeks) successfully attended by each participant.

### Outcome measures

The primary outcome was the Sialorrhea Clinical Scale for PD (SCS-PD), a validated 7-item scale assessing drooling severity. Scores range from 0 to 21, with higher scores indicating more severe symptoms (23).

Secondary outcomes included:

(1) UPDRS-II Item 6 (Sialorrhea): A clinician-rated scale from 0 to 4 (24).

(2) PD Self-Health Management Questionnaire: A 35-item scale assessing four dimensions: emotion (7 items), daily life (9 items), medical (9 items), and exercise management (10 items). Each item is rated on a 0–3 scale, with total scores ranging from 0 to 105; higher scores indicate superior self-health management capability (25).

### Statistical analysis

Statistical analysis was performed using IBM SPSS Statistics version 27.0. Continuous variables were expressed as mean ± standard deviation (SD). The normality of the data was verified using the Shapiro-Wilk test. As all randomized participants completed the study with no dropouts or loss to follow-up, data from all subjects were included in the final analysis. To compare post-intervention outcomes between groups while controlling for baseline imbalances, Analysis of Covariance (ANCOVA) was employed for both the primary outcome (SCS-PD) and all secondary outcomes (UPDRS-II item 6 and Self-Health Management scores), with baseline scores (T0) included as covariates. This approach was chosen to provide a more precise estimate of the treatment effect and minimize Type I error risk. For each outcome, we reported the adjusted mean difference, the 95% confidence interval (CI), and the standardized effect size (Cohen's *d*). Cohen's *d* was interpreted as small (0.2), medium (0.5), or large (0.8). A two-tailed *P* < 0.05 was considered statistically significant.

## Results

### Experts' general information

Following the invitation and screening process, 20 experts were initially engaged for the study (participation rate: 80.0%, 20/25). However, based on the completion of the subsequent consultation rounds, the final analysis was conducted on the 16 specialists who comprised the definitive Delphi panel. Representing nine provinces, the panel's diverse geographical distribution minimized regional bias, while its multidisciplinary composition ensured that the intervention framework was cross-validated through neurology and rehabilitation perspectives.

The 16 specialists possessed extensive professional experience, averaging 23.56 ± 9.50 years (range: 11–43), with a mean age of 47.50 ± 7.95 years (range: 39–64). All experts held high-ranking professional titles, including seven senior (43.75%) and nine associate senior titles (56.25%). In terms of education, 87.5% held a master's degree or higher (nine Master's, 56.25%; five PhDs, 31.25%). The panel comprised a core nursing group—including two nurse education specialists (12.5%), five nursing management specialists (31.25%), and five clinical nursing specialists (31.25%)—which was strategically complemented by specialists in neuropsychology (6.25%), neurorehabilitation (6.25%), neurodegenerative disorders (6.25%), and clinical nutrition (6.25%). Every expert involved had pertinent clinical experience with PD. Detailed basic information of the experts is presented in Table 1.

**Table 1:** Basic facts from experts (*N* = 16).

**Items**	**Number (%)**
**Age**	
< 40	1 (6.25)
40~50	12 (75.00)
>50	3 (18.75)
**Years of employment**	
< 20	5 (31.25)
20~30	8 (50.00)
>30	3 (18.75)
**Title**	
Associate senior	9 (56.25)
Senior professional	7 (43.75)
**Qualifications**	
Undergraduate	2 (12.50)
Master	9 (56.25)
Doctor	5 (31.25)
**Professional direction**	
Clinical nursing	5 (31.25)
Nursing management	5 (31.25)
Nursing education	2 (12.50)
Neurodegenerative diseases	1 (6.25)
Neurological rehabilitation	1 (6.25)
Neuropsychology	1 (6.25)
Clinical nutrition	1 (6.25)

### The level of expertise and zeal of professionals

An 80% response rate was achieved in the first round of expert consultation, with 20 surveys given and 16 valid questionnaires returned. All 16 valid questionnaires that were delivered in the second round were returned, yielding a 100% response rate. The first and second rounds of this study's *Cr* were 0.928 and 0.944, respectively.

### The degree of coherence and concentration of expert opinions

The items in the first round had mean significance scores ranging from 4.70 to 5.00, a Kendall's W of 0.166, and a *CV* between 0 and 0.109. With a Kendall's W of 0.375 and a *CV* ranging from 0 to 0.108, the mean significance ratings in the second round ranged from 4.63 to 5.00. The viability of everything in the professional correspondence. The feasibility score for each item in the first round had a Kendall's W of 0.137 and a coefficient of variation ranging from 0 to 0.150. In the second round, each item's feasibility score had a Kendall's W of 0.325 and a coefficient of variation ranging from 0 to 0.140. The results are displayed in Table 2.

**Table 2:** Kendall harmony coefficients and test results for two expert correspondence rounds.

**Rounds**	**Importance rating**	**χ^2^**	***P***	**Feasibility score**	**χ^2^**	***P***
1	0.166	193.888	< 0.001	0.137	160.016	< 0.001
2	0.375	336.000	< 0.001	0.325	291.200	< 0.001

### Findings from expert communication

After completing the initial round of expert contact, the study team included expert comments and recommendations into the related entries, followed by clinical work. (For specifics, see Table 3). The final draft of the intervention program for sialorrhea in PD patients was developed in the second round of correspondence with no objections from the experts. As indicated in Table 4, it included three primary indicators, 13 secondary indicators, and 39 tertiary indicators.

**Table 3:** The first round of delphi experts' advice and modification letters.

**Content**	**Expert opinion**	**Modification**
Delete content	1. The indicator “Behavioral Therapy” was deleted.	1. The items have been eliminated based on expert consensus.
	2. Delete “prone position” in item 2.4.2.	2. The items have been eliminated based on expert consensus.
Merge or change the content	1. Merge the indicators “anxiety” and “depression status” into “psychological care.”	1. The items have been consolidated based on expert consensus.
	2. Merge the indicators “improving self-management ability” and “enhancing quality of life.”	2. The items have been consolidated based on expert consensus.
	3. Two experts suggested merging identical entries under “swallowing disorder training.”	3. The items have been consolidated based on expert consensus.
	4. Three experts suggested merging identical entries under “oral function exercises.”	4. The items have been consolidated based on expert consensus.
	5. Add “slow walking, Tai Chi, square dancing, yoga, and other exercise methods” to entry 2.6.3.	5.2.6.3 Implement relaxation techniques including breathing exercises, guided imagery, slow walking, Tai Chi, square dancing, and yoga to ameliorate negative emotions in PD patients.
	6. Based on the opinions of multiple experts, include intervention strategies, intervention implementers, intervention frequency, intervention setting, intervention duration, and intervention format.	6. These elements have been incorporated based on expert consensus.
Modification content	1. Change the indicator “Self-Management” to “Enhancing Self-Management Skills.”	1.2.7 Enhancing Self-Management Skills.
	2. Five experts suggested that some secondary indicator items do not align with the dimension “Symptom Management Effectiveness” and recommended a revision.	2. Revisions have been made in accordance with expert recommendations.

**Table 4:** The SMT served as the foundation for the development of the final version of the intervention regimen for sialorrhea in PD.

**Intervention time, location, and format**	**Intervention protocol**	**Importance score ($\bar{\chi }$ ±*S*)**	***CV***	**Feasibility score ($\bar{\chi }$ ±*S*)**	***CV***
The first week, 15–20 min, Hospital and Community, Face-to-face interviews (focus group establishment)	1. Symptom experience	5.00	0	4.88 ± 0.34	0.07
	1.1 Symptom perception	5.00	0	5.00	0
	1.1.1 Examine the patient's quality of life, daily living activities, emotional condition, and social functioning. Ask them whether they feel that their present experiences and feelings are different from their typical baseline.	5.00	0	4.94 ± 0.25	0.05
The first week, 15–20 min, Hospital and Community, Face-to-face interviews (focus group establishment)	1.2 Symptom evaluation	5.00	0	4.75 ± 0.58	0.12
	1.2.1 Assess the ability of PD patients to describe the causes, beginning timing, progression, severity, and frequency of sialorrhea.	4.75 ± 0.45	0.09	5.00	0
	1.2.2 Evaluate the capacity of PD patients to control sialorrhea and its associated consequences.	4.81 ± 0.40	0.08	4.94 ± 0.425	0.05
	1.2.3 Assess PD patients' ability to explain how sialorrhea affects their quality of life.	4.75 ± 0.58	0.12	4.94 ± 0.25	0.05
The first week, 15–20 min, Hospital and Community, Face-to-face interviews (focus group establishment)	1.3 Symptom response	5.00	0	4.81 ± 0.54	0.11
	1.3.1 Examine the effects of sialorrhea on the behavior, quality of life, social and cultural adaption, psychological stability, and physical health of PD patients.	4.88 ± 0.34	0.07	4.88 ± 0.34	0.07
The first week, 15–20 min, Hospital and Community, Thematic lectures and handbook distribution	2. Symptom management strategies	5.00	0	4.75 ± 0.8	0.12
	2.1 Cognitive intervention	5.00	0	4.88 ± 0.34	0.07
	2.1.1 Explain the definition, causes, triggers, symptoms, progression, treatment options, and rehabilitative nursing training of PD.	4.94 ± 0.25	0.05	4.88 ± 0.50	0.10
	2.1.2 Have in-person conversations with PD patients to recognize and assist them in altering unfavorable attitudes and patterns of behavior.	4.88 ± 0.34	0.07	4.94 ± 0.25	0.05
	2.1.3 Inform PD patients about sialorrhea, the value of medication adherence, the advantages of leading a healthy lifestyle, rehabilitation training techniques, breathing relaxation techniques, and music therapy through multimedia, slides, videos, health education pamphlets, and health lectures.	4.81 ± 0.54	0.11	4.81 ± 0.54	0.11
	2.1.4 Determine the right intervention objectives for PD patients according to their illness stage (raise daily intervention time for patients in advanced stages as needed).	4.81 ± 0.40	0.08	4.88 ± 0.50	0.10
1–4 weeks,30 min per session, 5 sessions per week, Hospital and Community, Thematic lectures, hands-on demonstrations, and handbook distribution	2.2 Swallowing disorder training	5.00	0	4.75 ± 0.68	0.14
	2.2.1 Help PD patients relax their facial muscles by having them do exercises including inflating their cheeks, closing their eyes, opening and closing their mouths, smiling, and exhaling.	4.88 ± 0.50	0.10	5.00	0
	2.2.2 Teach PD patients how to stimulate the pharyngeal mucosa by creating ice cotton swabs. Additionally, instruct patients to put ice cubes in their mouths and alternately stimulate the posterior pharyngeal wall, tongue surface, and tongue arch with their tongues.	4.88 ± 0.34	0.07	4.94 ± 0.25	0.05
	2.2.3 To strengthen the swallowing reflex and improve swallowing function, instruct PD patients to alternatively stimulate the oropharynx with warm water, ice water, and lemon water.	4.81 ± 0.54	0.11	4.94 ± 0.25	0.05
	2.2.4 Instruct PD patients to repeatedly practice swallowing saliva and food.	4.94 ± 0.25	0.05	4.81 ± 0.54	0.11
1–4 weeks,30 min per session, 5 sessions per week, Hospital and Community, Thematic lectures, hands-on demonstrations, and handbook distribution	2.3 Oral function training	5.00	0	4.88 ± 0.50	0.10
	2.3.1 Help PD patients engage in oral muscular activities, including blowing, smiling, clenching teeth, opening and closing the mouth, bringing the tongue up, down, left, and right, and pursing the lips. Encourage patients to utilize a tongue retractor to improve their tongue's mixing ability and aid in tongue movement.	4.88 ± 0.50	0.10	4.88 ± 0.34	0.07
	2.3.2 Teach PD patients to practice chewing gum by completely chewing it with their teeth and tongue.	4.81 ± 0.54	0.11	4.88 ± 0.50	0.10
	2.3.3 Lead PD patients through soft palate exercises and voice training, such as singing exercises, Lee Silverman Voice Treatment (LSVT) voice training, and consonant articulation training. Pronouncing syllables like “b” and “p” can help patients' mouth muscles contract more. Give patients instructions to make the “a” or “ka” sounds while pressing their hands against a table.	4.88 ± 0.50	0.10	4.81 ± 0.54	0.11
	2.3.4 Lead PD patients through jaw movement exercises that strengthen the masseter muscles by moving the jaw in different directions.	5.00	0	4.81 ± 0.40	0.08
	2.3.5 Teach breathing techniques to PD patients, such as gradual exhalation and deep inhalation.	4.94 ± 0.25	0.05	4.88 ± 0.34	0.07
1–4 weeks, 15–20 min per session, once weekly, Hospital and Community, Thematic lectures, hands-on demonstrations, and handbook distribution	2.4 Posture adjustment	4.94 ± 0.25	0.05	4.75 ± 0.58	0.12
	2.4.1 Help PD patients practice sitting posture while keeping their shoulders and neck muscles relaxed and their posture straight.	4.94 ± 0.25	0.05	5.00	0
	2.4.2 To stop collected saliva from getting into the trachea, tell PD patients to sleep laterally.	4.81 ± 0.54	0.11	5.00	0
	2.4.3 Encourage people with PD to eat while seated, keeping their bodies symmetrical on both sides. To stop head tilting, use a chair that supports your head and back.	4.88 ± 0.50	0.10	4.88 ± 0.34	0.07
1–4 weeks, 15–20 min per session, once weekly, Hospital and Community, Health-themed lectures and healthy diet handbook distribution	2.5 Dietary guidance	5.00	0	4.94 ± 0.25	0.05
	2.5.1 Inform PD patients and their caregivers about healthy lifestyles and scientific dietary practices. Offer guidance on managing a healthy diet and disseminate relevant pamphlets and videos.	4.81 ± 0.40	0.08	4.81 ± 0.40	0.08
	2.5.2 Encourage PD patients to eat more fresh fruits and vegetables, add seafood in moderation, and permit modest amounts of tea, orange juice, and red wine.	4.88 ± 0.50	0.10	4.88 ± 0.34	0.07
	2.5.3 Help people with PD select healthy, light, and easily chewable foods. Eat less hard, salty, spicy, and protein-rich foods.	4.81 ± 0.54	0.11	4.75 ± 0.68	0.14
1–4 weeks, 15–20 min per session, once weekly, Hospital and Community, Face-to-face interviews	2.6 Psychological care	5.00	0	5.00	0
	2.6.1 Recognize the reasons behind PD sufferers' unpleasant feelings. Provide psychological counseling, listen empathetically to the causes of patients' bad emotions, and encourage patients to discuss their psychological states both before and after the disease start.	4.88 ± 0.34	0.07	4.94 ± 0.25	0.05
	2.6.2 Depending on the PD patients' tastes, play opera, mainstream music, folk tunes, light music, or calming music. Make sure the music has the right volume control and is calming and gentle.	4.81 ± 0.40	0.08	5.00	0
	2.6.3 To help PD patients deal with unpleasant emotions, try breathing exercises, guided visualization, yoga, Tai Chi, square dance, or slow strolling.	4.75 ± 0.58	0.12	4.75 ± 0.45	0.09
	2.6.4 Urge caregivers to give PD patients extra attention and support and to speak with them patiently.	5.00	0	4.94 ± 0.25	0.05
1–4 weeks, 15–20 min per session, once weekly, Hospital and Community, Thematic lectures and handbook distribution	2.7 Enhancing self-management skills	5.00	0	4.75 ± 0.58	0.12
	2.7.1 Give PD patients health education that is specific to their situation. To assist family members in overseeing the day-to-day health care of PD patients, family education should be strengthened.	5.00	0	4.88 ± 0.50	0.10
	2.7.2 Help PD patients with their medication, follow-ups, and doctor's appointments. Additionally, teach patients how to monitor themselves by noting, evaluating, and documenting their symptoms as well as any unfavorable or uncomfortable outcomes brought on by sialorrhea.	4.88 ± 0.34	0.07	4.81 ± 0.54	0.11
	2.7.3 Help people with PD manage their everyday tasks so they can continue to be independent.	4.88 ± 0.34	0.07	4.94 ± 0.25	0.05
	2.7.4 To maintain oral hygiene, teach PD patients to brush their teeth twice a day, rinse their mouths with saline after meals, and wash their faces with warm water and soft towels.	4.81 ± 0.40	0.08	4.94 ± 0.25	0.05
	2.7.5 To avoid soiled clothing and bedding, advise PD patients to keep towels under their pillows or chins when they are in a supine position. Wet garments, bedding, and pillows should be replaced right away.	4.88 ± 0.34	0.07	4.81 ± 0.54	0.11
	2.7.6 Offer nutritional recommendations based on PD patients' eating patterns and the findings of swallowing assessments.	4.63 ± 0.81	0.17	4.94 ± 0.25	0.05
1–4 weeks, 15–20 min per session, once weekly, Family, Telephone/video follow-up and home visits	3. Symptom management effectiveness	5.00	0	5.00	0
	3.1 Assessing symptom experience	5.00	0	4.94 ± 0.25	0.05
	3.1.1 Examine PD patients' symptom experiences by doing biweekly home visits and weekly phone/video follow-ups. Ascertain the patients' status with sialorrhea and ask if their symptoms and emotions have changed since the intervention.	5.00	0	4.88 ± 0.50	0.10
1–4 weeks, 15–20 min per session, once weekly, Family, Telephone/video follow-up and home visits	3.2 Evaluating symptom management effectiveness	4.63 ± 0.50	0.11	4.81 ± 0.54	0.11
	3.2.1 Assess if PD patients' quality of life, negative emotions, self-management abilities, and sialorrhea severity have improved.	4.81 ± 0.40	0.08	4.94 ± 0.25	0.05
1–4 weeks, 15–20 min per session, once weekly, Family, Telephone/video follow-up and home visits	3.3 Adjusting symptom management strategies	5.00	0	4.94 ± 0.25	0.05
	3.3.1 Use pictures, videos, and PowerPoint presentations to share information in a WeChat group on two to three subjects each week (risk factors, training for swallowing disorders, mouth muscle exercises, food advice, psychological care). Patients should be encouraged to update the group on their progress.	5.00	0	5.00	0
	3.3.2 Based on the PD patients' symptom experience and management efficacy, modify the care plan for the upcoming week. Adjust tactics using the intervention-feedback-revision process.	4.88 ± 0.34	0.07	5.00	0
	3.3.3 Evaluate PD patients' compliance and whether their objectives have been met. Patients who exhibit great compliance or who have accomplished their objectives should be commended and given tangible incentives. A thorough assessment was carried out to determine the root causes of patients' poor adherence or inability to meet treatment objectives, and then the management approach was modified as necessary.	4.75 ± 0.58	0.12	4.94 ± 0.25	0.05

### Recruitment and retention

**Figure 2:**
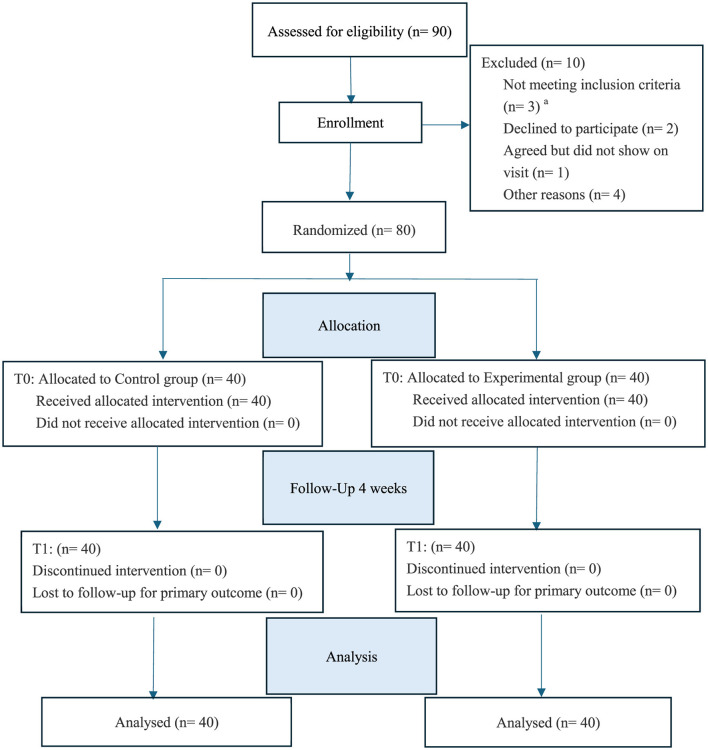
CONSORT flow diagram. ^a^The three participants who did not meet the inclusion criteria were excluded for the following reasons: one participant was taking medication that affects saliva secretion, and the other two participants were currently enrolled in a separate clinical trial to avoid confounding effects.

The study assessed a total of 90 potential participants for eligibility. Ten individuals were excluded due to not meeting the inclusion criteria (*n* = 3), declining to participate (*n* = 2), agreeing but failing to show up for the visit (*n* = 1), and other reasons (*n* = 4). Consequently, 80 eligible participants were enrolled and randomly allocated. At baseline (T0), participants were equally assigned to the Control group (*n* = 40) and the Experimental group (*n* = 40). Notably, all participants in both arms successfully received the allocated intervention (Control group *n* = 40, Experimental group *n* = 40), with no dropouts prior to intervention commencement. During the 4-week follow-up period (T1), both groups maintained a 100% retention rate, as no participants discontinued the intervention or were lost to follow-up for the primary outcome. Therefore, data analysis was conducted on the full sample size (*n* = 40) for both groups (see Figure 2).

### Feasibility and safety outcomes

Regarding feasibility, the recruitment rate was 92.0% (80/87 eligible patients). Of the 90 patients originally screened, 10 were excluded prior to randomization (comprising 3 ineligible candidates and 7 eligible candidates who declined, failed to attend, or had other reasons), yet all 80 enrolled participants completed the full 4-week study and follow-up, resulting in a 100% retention rate. Adherence to the intervention protocol was high; the session completion rate was 97.5% in the experimental group, with only two participants missing more than one session due to personal scheduling conflicts. Acceptability was high, as indicated by qualitative feedback from patients who found the oral exercises easy to integrate into their daily routines. Regarding safety, no intervention-related adverse events, such as choking, aspiration, or oral muscle strain, were reported during the study period.

### Baseline characteristics of participants

At baseline, a total of 80 eligible participants were enrolled and randomized. The two groups' initial characteristics were well- balanced. The experimental group's mean age was (68.28 ± 7.17) years, while the control group's was (68.25 ± 7.22) years. There were 17 men and 23 women in the experimental group, compared to 21 men and 19 women in the control group. These demographic data did not show any statistically significant differences between the groups (*P* > 0.05) (Table 5).

**Table 5:** Comparison of baseline characteristics between the two groups (*N* = 80).

**Items**	**Experimental group (*n* = 40)**	**Control group (*n* = 40)**	**Test statistic**	***P***
Age (years,χχ ±*s*)	68.28 ± 7.17	68.25 ± 7.22	−0.016^b^	0.988
Sex [*n* (%)]			0.802^a^	0.370
Male	17 (42.50%)	21 (52.50%)		
Female	23 (57.50%)	19 (47.50%)		
Ethnicity [*n* (%)]			0.480^e^	0.488
Han	39 (97.50%)	40 (100.00%)		
Other	1 (2.50%)	0 (0.00%)		
Marital status [*n* (%)]			0.424^a^	0.515
Unmarried/Divorced/Widowed	5 (12.50%)	7 (17.50%)		
Married	35 (87.50%)	33 (83.00%)		
Living arrangement [*n* (%)]			4.397^c^	0.161
Living alone	0 (0.00%)	2 (5.00%)		
Living with spouse	33 (82.50%)	33 (82.50%)		
Living with children	7 (17.50%)	4 (10.00%)		
Other	0 (0.00%)	1 (2.50%)		
Education level [*n* (%)]			−1.768^d^	0.770
Primary school or below	16 (40.00%)	11 (27.50%)		
Junior high school	13 (32.50%)	15 (37.50%)		
Senior high school/ Technical school	11 (27.50%)	10 (25.00)		
College or above	0 (0.00)	4 (10.00)		
Payment method for medical expenses [*n* (%)]			—	0.830
Self-paid	8 (20.00%)	6 (15.00%)		
Government-funded	0 (0.00%)	0 (0.00%)		
Medical insurance	32 (80.00%)	34 (85.00%)		
Monthly income (RMB) [*n* (%)]			1.946^a^	0.584
< 2,000	13 (32.50%)	10 (25.00%)		
2,000–5,000	19 (47.50%)	20 (50.00%)		
>5,000	8 (20.00%)	10 (25.00%)		
Hoehn and Yahr stage			−0.315^d^	0.752
Early stage (Stage 1–1.5)	13 (32.50%)	10 (25.00%)		
Middle stage (Stage 2–3)	24 (60.00%)	28 (70.00%)		
Late stage (Stage 4–5)	3 (7.50%)	2 (5.00%)		
Disease duration (years) [M (P_25_, P_75_)]	4.00 (2.00, 6.75)	4.00 (2.00, 5.75)	−0.185	0.853

^*a*^Pearson's chi-square test; ^*b*^*t-*test; ^*c*^Fisher's exact test; ^*d*^Kruskal-Wallis test; ^*e*^Continuity correction test, the test statistic for the remaining test is the *U*-value.

### Effectiveness of the SMT-based Intervention on sialorrhea and self-management

At baseline, no statistically significant differences were observed between the two groups in sialorrhea severity (UPDRS-II or SCS-PD) or self-management scores (*P* > 0.05). After the 4-week intervention, ANCOVA adjusted for baseline values revealed significant between-group differences favoring the experimental group. Specifically, the experimental group showed a significantly lower SCS-PD score compared with the control group (adjusted mean difference = −2.34; 95% CI: −3.98 to −0.70; *P* = 0.012), with a medium effect size (Cohen's *d* = 0.51). Significant differences were also observed in UPDRS-II item 6 scores (adjusted mean difference = −0.36; 95% CI: −0.65 to −0.07; *P* = 0.016; Cohen's *d* = 0.55) and self-management ability (adjusted mean difference = 5.47; 95% CI: 0.38 to 10.56; *P* = 0.036; Cohen's *d* = 0.48). Detailed results are presented in Table 6.

**Table 6:** Comparison of study outcomes between the two groups at baseline and 4-week follow-up (*N* = 80).

**Items**	**Group**	**Baseline (T0)**	**4-Weeks Post (T1)**	**Adjusted mean diff. (95% CI)^*a*^**	***P*-value^*b*^**	**Cohen's *d***
UPDRS-II (6)	Exp. (*n* = 40)	1.83 ± 0.78	1.40 ± 0.71	−0.36	0.016^*^	0.55
	Ctrl. (*n* = 40)	1.85 ± 0.83	1.78 ± 0.89	(−0.65, −0.07)		
SCS-PD	Exp. (*n* = 40)	11.60 ± 4.01	9.13 ± 3.20	−2.34	0.012^*^	0.51
	Ctrl. (*n* = 40)	11.08 ± 3.90	10.95 ± 3.92	(−3.98, −0.70)		
Self-management Ability	Exp. (*n* = 40)	42.68 ± 10.64	48.23 ± 10.06	5.47	0.036^*^	0.48
	Ctrl. (*n* = 40)	43.15 ± 10.84	43.23 ± 10.84	(0.38, 10.56)		

UPDRS-II (6), Sialorrhea item of the Unified Parkinson's Disease Rating Scale Part II; SCS-PD, Sialorrhea Clinical Scale for Parkinson's Disease. ^*a*^Adjusted Mean Difference (95% CI) and ^*b*^P-values were derived from ANCOVA models using baseline scores as covariates. ^*^P < 0.05.

## Discussion

### The necessity and urgency of creating an SMT-based sialorrhea therapeutic program for PD patients

The incidence of PD has been steadily increasing due to the world' aging population, making it a major global public health concern (26). A prevalent non-motor symptom of PD, sialorrhea not only negatively affects patients' quality of life but also sets off a number of clinical and psychological issues. Sialorrhea can physiologically result in impaired speech and trouble eating, and prolonged saliva exposure may cause infection and soreness in the perioral skin (8). Psychosocially, sialorrhea patients frequently struggle with communication, emotional distress, embarrassment, and a lack of social support (27).

Nursing interventions have been shown to be successful in enhancing cognition, mood, and quality of life in older PD patients as well as reducing the disease's development, despite the drawbacks or restrictions of current pharmaceutical and injectable treatments (18). A patient-centered nursing strategy that allows patients to actively cope is desperately needed, given the complexity of sialorrhea, its multiple impact, and the limitations of existing therapies. SMT provides the perfect foundation for this, focusing on multi-modal therapies, systematic assessment, and ongoing evaluation to effectively control symptoms.

Thus, it is very important to develop a nurse intervention program based on SMT. In order to create customized and dynamic management plans, this program focuses on the patient's experience of sialorrhea symptoms and uses non-invasive, low-side-effect structured management techniques. In addition to empowering patients to actively manage sialorrhea and the distress it causes, this strategy may improve quality of life, lessen complications, alleviate symptoms at their source, and ultimately offer a successful route toward more thorough and compassionate care for PD patients.

### The SMT-based intervention program for PD sialorrhea is scientifically rigorous and expert-validated

Using a literature review and two rounds of Delphi expert consultation, this study created an intervention program for sialorrhea in PD patients based on SMT, proving its significance and urgency. To ensure representativeness and authority, 16 highly qualified professionals from academia and clinical practice were chosen for the Delphi consultation. According to the results of the expert consultation, all of the *Cr* were greater than 0.7, which suggests that they are very credible (28). All of the variation coefficients were ≤ 0.25, and Kendall's W was 0.375, indicating strong expert unanimity and demonstrating the scientific validity of the consultation results.

### The SMT-based sialorrhea therapeutic approach for PD patients is flexible and dynamic

Significant variation in clinical presentations of sialorrhea in PD patients results from the interaction of several factors, including living environment, drug response, and disease progression. This implies that the intervention program must be dynamically adaptive.

Intervention strategies are tailored based on each patient's unique symptom experience. While the total daily session duration remains fixed at 30 min, the intensity and focus of the exercises are dynamically adjusted in accordance with the patient's progress and feedback. The nursing team conducts regular assessments to monitor changes in sialorrhea severity and the patient's subjective experience, allowing for timely optimization of the management plan.

To address the multidimensional nature of sialorrhea (encompassing both physiological and psychological aspects), this project employs a nurse-led intervention framework grounded in multidisciplinary protocols (designed with input from neurologists, rehabilitation therapists, and psychologists). Rather than providing continuous hospital-based therapy, the nurses act as educators and supervisors to empower patients with the skills needed for home-based self-management. This approach covers swallowing techniques, oral motor exercises, and psychosocial support, ensuring that patients can effectively integrate these management strategies into their daily living environments.

### The generalizability and sustainability of a SMT-informed sialorrhea intervention program for PD patients

We recognize that there are inherent challenges in implementing such a program, which are made worse by low household income and a lack of long-term monitoring and expert advice. Because they lack the necessary skills or motivation, non-professional family caregivers may find it difficult to carry out the intervention effectively, which could result in poor adherence and less than ideal results. Therefore, the development of this program places a high priority on streamlining operational procedures and promoting non-pharmacological interventions that are affordable, simple to learn, and doable at home. Examples of these interventions include modified posturing, targeted swallowing exercises, regular oral hygiene, and basic tongue and facial muscle training. Additionally, through programs like community health education, remote medical support, and the creation of succinct graphical aids, we will fully empower patients and their family caregivers. Quantitative tracking of intervention fidelity was maintained through a standardized nursing manual. The program demonstrated high feasibility, with a recruitment rate of 92.0% and an intervention adherence rate of 97.5%. The primary implementation barrier identified was the logistical challenge for elderly patients to maintain regular swallowing frequency cueing in distraction-heavy home environments. Despite these challenges, the high retention rate suggests the program is acceptable to the PD population. This multifaceted strategy seeks to maximize symptom control with little resources, guaranteeing the program's long-term effectiveness and wide application.

### Efficacy and applicability of a sialorrhea intervention program for PD patients based on SMT

The primary findings of this pilot study demonstrate that a structured intervention grounded in SMT significantly alleviates sialorrhea severity and enhances self-management capabilities in patients with PD. By integrating individualized instruction, the program moves beyond passive symptom suppression, enabling patients to comprehend the causes and consequences of their symptoms. This approach fosters active self-evaluation and mastery over the illness, which is reflected in the significantly higher self-management scores observed in the experimental group (*P* < 0.05). The reliability of these improvements is statistically bolstered by our use of ANCOVA to adjust for baseline imbalances, ensuring that the observed gains are directly attributable to the intervention components—such as targeted oral-motor exercises and SMT-based counseling—rather than random baseline variation.

Beyond statistical significance, the clinical relevance of these effects is underscored by the magnitude of improvement. The observation of medium effect sizes (Cohen's *d* ranging from 0.48 to 0.55) suggests that the program provides substantial clinical benefits. While a universal consensus on the Minimal Clinically Important Difference (MCID) for the SCS-PD scale is still emerging, previous literature suggests that a 2-point reduction or a 20% change from baseline represents a meaningful improvement for patients (29, 30). In the present study, the experimental group achieved an average reduction of 2.47 points (a 21.3% improvement), exceeding this critical threshold. In practical terms, this translates to a reduced daily reliance on handkerchiefs and a decrease in social embarrassment, directly enhancing the patients' willingness to engage in social communication.

When placed within the broader context of existing treatments, this nurse-led program occupies a unique clinical niche. While pharmacological interventions, such as botulinum toxin injections, often yield larger immediate effect sizes (Cohen's *d* > 0.8), they are frequently limited by their invasive nature and the risk of side effects like dysphagia or xerostomia (31, 32). In contrast, our approach emphasizes sustainable self-management. Compared to traditional speech and swallow therapy programs that rely heavily on professional-led clinical sessions, the SMT framework promotes long-term adherence in home-based settings. Furthermore, while common behavioral strategies like ‘swallow-on-cue' focus primarily on physical mechanics, our program uniquely integrates these with psychosocial support. This addresses the emotional distress and physical discomforts, such as perioral skin damage, that purely motor-based training may overlook.

### Limitations

#### Study design and generalizability

Firstly, this study utilized a single-center design with a relatively small sample size, which may limit the generalizability (external validity) of our findings to more diverse clinical settings or broader patient populations. While this research represents the initial foundational phase of an ongoing program, future multi-center studies with larger, more diverse cohorts are required to further validate these preliminary results.

(1) Duration and long-term follow-up

The intervention period was relatively short, and the investigation lacked long-term follow-up assessments. Consequently, the sustained efficacy and long-term effects of the intervention regimen on sialorrhea remain unconfirmed. Future research should incorporate longitudinal tracking to determine whether the observed improvements are maintained over extended periods.

(2) Bias and blinding

Another important limitation is the absence of true participant blinding, which is often challenging in behavioral or non-pharmacological interventions. This lack of blinding increases the risk of performance bias and reporting bias, as participants' awareness of their group assignment may have influenced their self-reported outcomes.

(3) Outcome measures

The assessment of sialorrhea relied primarily on subjective scales. The lack of objective drooling measures (such as saliva collection or swab weighing) may affect the precision of our data. Additionally, as a pilot trial focused on feasibility and immediate impact, this report did not include broader patient-centered outcomes, such as Quality of Life (QoL) or caregiver burden. Although these dimensions are being integrated into the current expanded phase of our research (using instruments like the PDQ-39 and Zarit Caregiver Burden Interview), their absence in this preliminary report limits our understanding of the program's full psychosocial benefits.

(4) Statistical and confounding factors

Finally, although we employed ANCOVA to adjust for baseline differences, our statistical analysis did not include corrections for multiple comparisons (such as Bonferroni correction) across the multiple primary and secondary outcomes, which may increase the risk of Type I errors. Furthermore, we acknowledge potential confounding due to medication changes; while participants were encouraged to maintain stable medication, fluctuations in concurrent PD treatments were not strictly controlled or analyzed. Future studies should employ more robust statistical modeling, such as linear mixed-effects models, and incorporate more stringent medication monitoring to account for these variables.

## Conclusions

In this study, an SMT-based sialorrhea therapeutic program for PD patients was developed through a comprehensive literature review and two rounds of Delphi expert consultation. A structured three-tiered indicator system, comprising three primary, 13 secondary, and 39 tertiary indicators, was established to guide the intervention. While the program is ultimately designed to enhance patients' long-term quality of life, the current study focused on its immediate impact on self-health management skills and the alleviation of sialorrhea-related distress—both of which are critical determinants of patient well-being. Preliminary findings demonstrate that this approach effectively bridges theoretical frameworks with clinical nursing practice. These results provide encouraging evidence to support the feasibility of the program and lay the groundwork for future large-scale trials aimed at standardizing protocols for symptom control and holistic patient care.

## Data Availability

The original contributions presented in the study are included in the article/supplementary material, further inquiries can be directed to the corresponding authors.
